# Feasibility of using remotely delivered Spring Forest Qigong to reduce neuropathic pain in adults with spinal cord injury: a pilot study

**DOI:** 10.3389/fphys.2023.1222616

**Published:** 2023-08-31

**Authors:** Ann Van de Winckel, Sydney T. Carpentier, Wei Deng, Lin Zhang, Angela Philippus, Ricardo Battaglino, Leslie R. Morse

**Affiliations:** ^1^ Division of Physical Therapy, Division of Rehabilitation Science, Department of Rehabilitation Medicine, Medical School, University of Minnesota, Minneapolis, MN, United States; ^2^ Division of Rehabilitation Science, Department of Rehabilitation Medicine, Medical School, University of Minnesota, Minneapolis, MN, United States; ^3^ Division of Biostatistics, School of Public Health, University of Minnesota, Minneapolis, MN, United States; ^4^ Department of Rehabilitation Medicine, Medical School, University of Minnesota, Minneapolis, MN, United States

**Keywords:** spinal cord injury, neuropathic pain, body awareness, autonomic function, Qigong, spasm, mood

## Abstract

**Introduction:** Approximately 69% of 299,000 Americans with spinal cord injury (SCI) suffer debilitating chronic neuropathic pain, which is intractable to treatment. The aim of this study is to determine feasibility, as the primary objective, and estimates of efficacy of a remotely delivered Qigong intervention in adults with SCI-related neuropathic pain, as the secondary objective.

**Methods:** We recruited adults with SCI-related neuropathic pain, with SCI ≥3 months, with complete or incomplete SCI, and highest neuropathic pain level of >3 on the Numeric Pain Rating Scale (NPRS), using nationwide volunteer sampling. Using a non-randomized controlled trial design, participants practiced Spring Forest Qigong’s “Five Element Qigong Healing Movements” (online video) by combining movement to the best of their ability with kinesthetic imagery, at least 3x/week for 12 weeks. Adherence was automatically tracked through the Spring Forest Qigong website. Outcomes of neuropathic pain intensity (NPRS) were assessed weekly, and SCI-related symptoms were assessed at baseline, 6, and 12 weeks of Qigong practice and at 6-week and 1-year follow-ups.

**Results:** We recruited 23 adults with chronic SCI (7/2021–2/2023). In total, 18 participants started the study and completed all study components, including the 6-week follow-up. Twelve participants completed the 1-year follow-up assessment. Feasibility was demonstrated through participants’ willingness to participate, adherence, and acceptability of the study. Mean age of the 18 participants was 60 ± 12 years, and they were 15 ± 11 years post-SCI with the highest baseline *neuropathic pain* of 7.94 ± 2.33, which was reduced to 4.17 ± 3.07 after 12 weeks of Qigong practice (Cohen’s *d* = 1.75). This pain relief remained at 6-week and 1-year follow-ups. Participants reported reduced spasm frequency (change score 1.17 ± 1.20, *d* = 0.98) and severity (0.72 ± 1.02, *d* = 0.71), reduced interference of neuropathic pain on mood (3.44 ± 2.53, *d* = 1.36), sleep (3.39 ± 2.40, *d* = 1.41), daily activities (3.17 ± 2.77, *d* = 1.14), greater ability to perform functional activities (6.68 ± 3.07, *d* = 2.18), and improved mood (2.33 ± 3.31, *d* = 0.70) after Qigong.

**Discussion:** Remote Spring Forest Qigong’s “Five Element Qigong Healing Movements” practice is feasible in adults with SCI-related neuropathic pain, with promising prolonged results of neuropathic pain relief and improvement in SCI-related symptoms after Qigong practice.

**Clinical trial registration:** https://www.clinicaltrials.gov/ct2/show/NCT04917107, identifier NCT04917107

## 1 Introduction

Chronic neuropathic pain is described as sharp, shooting, stabbing, electric, or burning, and sometimes excruciating pain. The mechanisms underlying neuropathic pain are not completely understood but are commonly reported as being related to damage to the somatosensory system in the peripheral or central nervous system (Raja et al., 2020; Ghazisaeidi et al., 2023). Due to this damage, changes occur at all levels of somatosensory circuitry (i.e., the spinal cord, brainstem, and brain), altering the sensorial communication between the brain and the body, resulting in the development of various degrees of sensory and motor impairment and aberrant pain sensations above, below, or at the level of the lesion (Chambel, Tavares, and Cruz, 2020; Forte et al., 2022). Furthermore, changes in brain function may contribute to neuropathic pain (Hawasli et al., 2018; Huynh et al., 2019; Black et al., 2021).

Chronic neuropathic pain occurring in approximately 69% of the 299,000 Americans with spinal cord injury (SCI) is debilitating and persistent, and it is the most intractable type of chronic pain ( Guy et al., 2016; Hatch et al., 2018; Raja et al., 2020; Leitzelar and Koltyn, 2021 ). Accessible treatment options (exercise and medication) have limited success in reducing pain (Kramer et al., 2017; Hatch et al., 2018; Kim et al., 2020; Felix et al., 2022; National Spinal Cord Injury Statistical Center, 2022). For instance, pain medications result in <50% pain reduction for only approximately 1/3 of the people trying them and some with notable adverse events. Studies conducted with SCI stakeholders confirm that accessible treatments are limited; that pain medications insufficiently relieve pain but carry high risks for addiction and adverse effects; and that improved patient access to non-pharmacological approaches for neuropathic pain is urgently needed (Guy et al., 2016; Hatch et al., 2018; Leitzelar and Koltyn, 2021).

Concurrently with reduced or absent sensation after SCI, accumulating evidence shows that adults with SCI experience body awareness deficits (Lenggenhager et al., 2012; Scandola et al., 2017; Kaur et al., 2019; Leemhuis et al., 2019; Osinski et al., 2020; De Martino et al., 2021; Leemhuis et al., 2021; Moro et al., 2021; Vázquez-Fariñas and Rodríguez-Martin, 2021; Leemhuis et al., 2022; Maggio et al., 2022; Moro et al., 2022; Vastano, Costantini, and Widerstrom-Noga, 2022; Vastano and Widerstrom-Noga, 2023), which is thought to contribute to the production and maintenance of chronic neuropathic pain (Lenggenhager et al., 2012; Leemhuis et al., 2019; Leemhuis et al., 2022; Vastano et al., 2022). Body awareness refers to an attentional focus on and awareness of internal body sensations, including awareness of how the body/body parts are positioned and move in space (Mehling et al., 2011).

Mind and body approaches improve body awareness and thus could be a viable approach to treating neuropathic pain (Impett et al., 2006; Mehling et al., 2011; Naranjo and Schmidt, 2012; Schmalzl et al., 2014; Jong et al., 2016; Mehling et al., 2018). However, research demonstrating the effectiveness of mind and body approaches for reducing pain in SCI is limited to seven studies among which only two (yoga and Tai Chi) (Shem et al., 2016; Curtis et al., 2017; Chalageri et al., 2021) reported pain reduction but without mentioning the type of pain (Tsang et al., 2015; Shem et al., 2016; Curtis et al., 2017; Qi et al., 2018; Madhusmita and Ebnezar, 2019; Hearn and Cross, 2020; Chalageri et al., 2021). Mind and body research in SCI is recent, and often movement modifications are needed to allow participation by adults with SCI. Chalageri et al. (2021) reported pain reduction after yoga meditation combined with conventional rehabilitation, mostly benefitting adults with acute SCI and paraplegia (Chalageri et al., 2021). Curtis et al. (2017) reported greater improvements in depressive symptoms and self-compassion, but not pain, in adults with SCI practiced adaptive yoga vs. waitlist group (Curtis et al., 2017). A seated Tai Chi program was well tolerated in adults with SCI with benefits in pain, emotional sense, and physical sense of wellbeing after each session, but the weekly in-person classes had a dropout rate of 60% (Shem et al., 2016). Tsang et al. (2015) reported greater improved dynamic sitting balance and grip strength after 12 weeks of sitting Tai Chi in 11 adults with SCI vs. eight controls (Tsang et al., 2015). With the exception of Chalageri et al.’s study (*n* = 91), sample sizes were small (*n* = 23, *n* = 26, and *n* = 19), and only Tsang et al. (2015) included adults with incomplete tetraplegia; all others recruited adults with paraplegia only. Thus, studies with accessible interventions for adults with tetraplegia are needed. Of the mind and body approaches, Qigong seems the most accessible approach for adults with SCI due to the simple, gentle movements, combined with a focus on breathing and body awareness. Remote Qigong delivery provides a feasible approach for adults with SCI as it eliminates reported in-person intervention barriers of transportation and scheduling difficulties (Huberty et al., 2017).

Therefore, our primary objective was to determine the feasibility of a 12-week Qigong practice with a remotely delivered Spring Forest Qigong “Five Element Healing Movements” video in adults with SCI-related neuropathic pain. Qigong could be practiced in standing, sitting, or lying positions. Our Qigong practice combined actively moving to the participant’s ability level with kinesthetic imagery, i.e., focusing on the feeling of moving the whole body *as if* in an upright position, because imagery may be an additional way to improve body awareness and reduce pain (Scandola et al., 2017; Kaur et al., 2019). Therefore, adults with high-level tetraplegia after SCI could practice Qigong in their power wheelchair or lying down. In contrast, in other studies, the requirement of minimum active muscle strength to perform the exercises often excludes them. Our secondary objective was to calculate estimates of the efficacy of Qigong practice in adults with SCI-related neuropathic pain to inform future efficacy clinical trials.

## 2 Materials and methods

### 2.1 Study design and recruitment

Full detail of the protocol can be found in Van de Winckel et al. (2023a). We used nationwide volunteer sampling through fliers and announcements on relevant websites, in the community, through the M Health/Fairview healthcare recruitment system, and in locations within the Minnesota Regional Spinal Cord Injury Model System (MN SCIMS). More specifically, we recruited from all clinical site partners within the MN SCIMS, which include hospitals, clinics, and rehabilitation centers in the *Twin Cities* and Mayo Clinic in Rochester. We have ongoing collaborations within the Twin Cities with the UMN Medical Center, M Health/Fairview; MN SCI associations; Regions Hospital; Courage Kenny Rehabilitation Institute; Get Up Stand Up to Cure Paralysis; Unite2Ffight Paralysis, and Fit4Recovery. Healthcare providers provided enrollment information and materials to potential participants and posted fliers in their locations. M Health/Fairview had a recruitment system in place where approximately 1,000 letters were sent from the electronic medical record system directly to patients with SCI and neuropathic pain. Patients who were interested in participating contacted the researchers. This was a very successful method of recruitment. Furthermore, we received calls and emails from potential participants from all over the United States when they saw study information from professional websites displaying information on the studies (through fliers or interview) or when they found the study through ClinTrial.gov.

### 2.2 Participants

We recruited adults with complete or incomplete SCI ≥3 months, medically stable, and with the highest neuropathic pain level of >3/10 on the Numeric Pain Rating Scale (NPRS) (Hanley et al., 2006) who were willing to participate in a 12-week Remote Qigong practice (mind-body approach), fluent in English, and had access to the internet and a computer/iPad or smartphone.

The study was conducted in accordance with the Declaration of Helsinki principles (2013) (WMA Declaration of Helsinki–Ethical Principles for Medical Research Involving Human Subjects, 2021). The study was approved by the Institutional Review Board (IRB) of the University of Minnesota (IRB# STUDY00011997). The CONSORT reporting guidelines were followed (Moher et al., 2010; Boutron et al., 2017). After signing HIPAA/informed eConsent, study staff members acquired demographic information, general health, medical history, screened for cognitive impairments (Mini-Mental State Examination-short version, cutoff score <13/16) (Folstein, Folstein, and McHugh, 1975; Cummings, 1993), and for kinesthetic motor imagery ability (Kinesthetic and Visual Imagery Questionnaire, cutoff score <15/25 points) (Malouin et al., 2007). Data were collected on REDCap, which uses a MySQL database via a secure web interface.

### 2.3 Intervention

**FIGURE 1:**
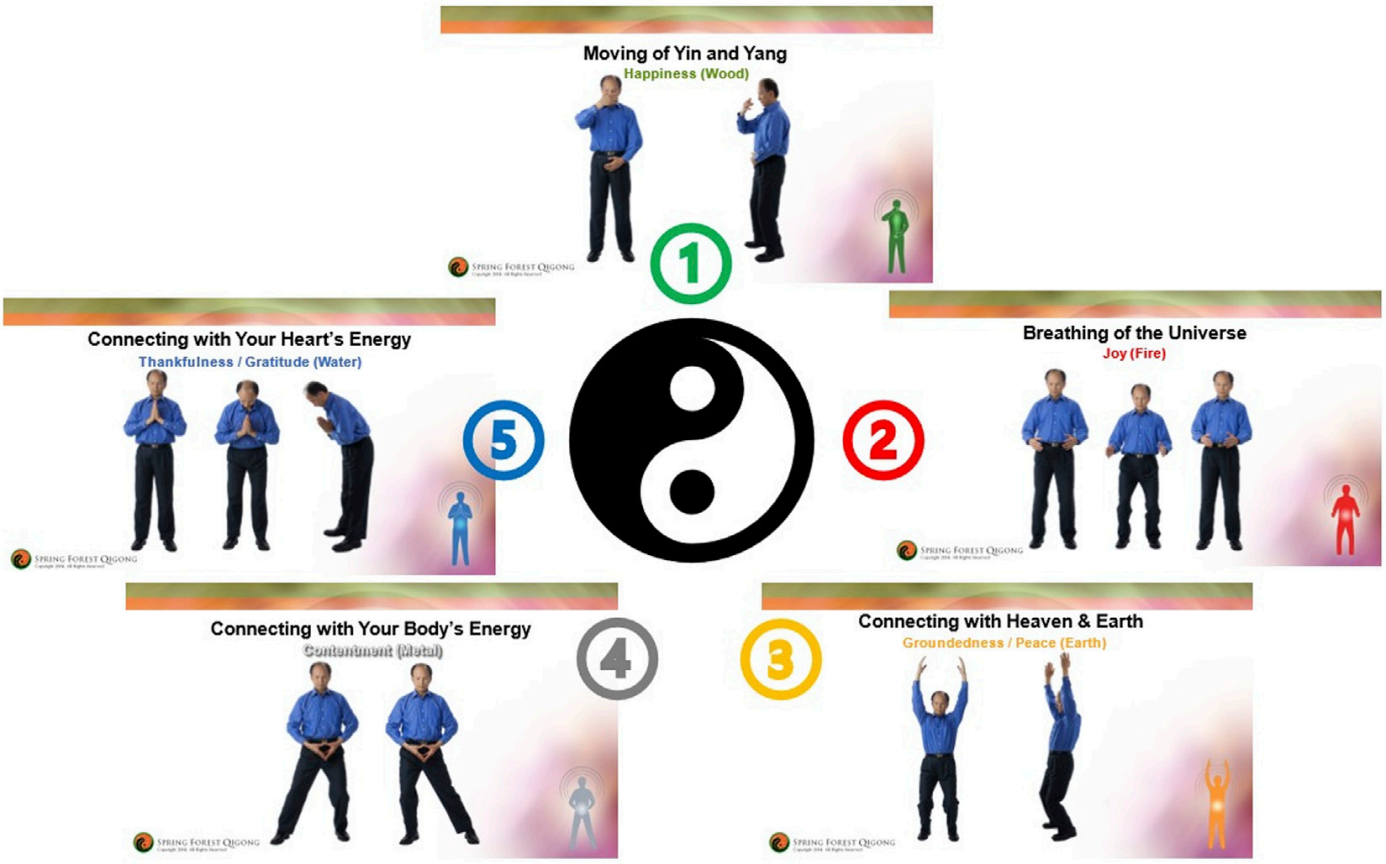
Spring Forest Qigong’s “Five Element Qigong Healing Movements”. The five movements are presented in clockwise order. Movement 1: moving of Yin and Yang; movement 2: breathing of the universe; movement 3: connecting with the heaven and Earth; movement 4: connecting with your body’s energy; and movement 5: connecting with your heart’s energy. Details of how the different movements are performed are presented in Van de Winckel et al. (2023a).

We used the Spring Forest Qigong’s “Five Element Healing Movements” video (41 min), in which Grand Master Lin demonstrates five gentle horizontal and vertical arm and leg movements in specific postures in the standing position. A Qigong Master (Spring Forest Qigong Center, Minnesota) taught the 6-h introductory class over Zoom. Participants accessed the video with a study number and password and were asked to practice at least 3x/week for 12 weeks in any location of their choice with an internet connection. Figure 1 shows the five Qigong movements. More details are available in Van de Winckel et al. (2023a). Participants were instructed to actively move along with the video however much they comfortably could and to perform kinesthetic imagery at the same time. The first author developed specific kinesthetic imagery instructions to allow participants with all levels of mobility to participate maximally in the Qigong practice: participants were asked to focus on the feeling of the body as if they were standing up (regardless of whether they were actually standing, sitting, or lying down) and to imagine the *feeling* of the soles of their feet being in contact with the floor and the *feeling* of the flow while imagining performing the whole-body movements, rather than to “visualize” the whole-body movement. If reproducing this feeling was difficult, participants were asked to associate positive memories from before their SCI with this imagined standing posture, i.e., the feeling of warm sand under the soles of their feet when walking on the beach.

During the 6-week follow-up, participants were not practicing Qigong, but afterward, participants could restart their Qigong practice at the frequency of their choice so we could evaluate behavioral changes, neuropathic pain, and function at 1-year follow-up.

### 2.4 Outcome measures

#### 2.4.1 Primary outcome

Based on prior literature (Rounsaville, Carroll, and Onken, 2001; Czajkowski et al., 2015; Winckers et al., 2015; Greenberg et al., 2020; 2019; Carl et al., 2020), we assessed the following *a priori* feasibility indicators for our primary objective: recruitment estimate of 40% of adults with complete SCI and 60% of adults with incomplete SCI; Qigong adherence of minimum 70% of participants practicing at least 2x/week; maximum 30% attrition; none of the questionnaires fully missing in >25% of the participants; mild study-related adverse events in maximum 10% of participants; and ≥70% participants satisfied with the program. Quotes from participants were used to identify satisfaction with the Qigong practice and the study. Video access (day, time, and duration) was automatically tracked via the Spring Forest Qigong website, and the logs were provided to the first author. The first author, a certified Spring Forest Qigong practice group leader, level 5 of 5 in the Spring Forest Qigong with Qigong practice experience, organized weekly 1:1 check-ins to address questions, perceptions, effects, and satisfaction with the Qigong practice, demonstrate movements if needed, and monitor adverse events. Given the slow, gentle movements, kinesthetic imagery, and weekly check-ins, we considered Qigong practice *risks* minimal and limited to mild transient discomfort. Participants continued with regular healthcare appointments and neuropathic pain medication if needed, but other health appointments for neuropathic pain (e.g., osteopathy) were not permitted during the study to avoid concomitant effects.

#### 2.4.2 Secondary outcomes

As estimate of efficacy outcomes for larger future randomized controlled trials, graduate students called participants weekly to monitor the highest, average, and lowest *neuropathic pain* intensity ratings (NPRS) (Hanley et al., 2006). Distinctions between types of pain were made using the National Institute of Neurological Disorders-Common Data Elements (NINDS-CDE) International SCI Pain Basic Data Set Version 2.0 (Widerström-Noga et al., 2014). The graduate students noted neuropathic pain medication dosage taken that week, recent illnesses, and healthcare utilization, including recent hospitalizations.

Other outcome measures were collected by graduate students over Zoom at five time points: baseline, 6 weeks (mid-Qigong practice), 12 weeks (end of Qigong practice), 6-week follow-up, and 1-year follow-up.

The NINDS-CDE International SCI Pain Basic Data Set Version 2.0 (Widerström-Noga et al., 2014) also assessed pain dimensions (e.g., pain location) and how intense pain interferes with mood, activity, and sleep. We evaluated the frequency of spasms and spasm severity (Penn Spasm Frequency Scale) (Mills et al., 2018); mood (Patient Health Questionnaire-9, PHQ-9) (Kroenke, Spitzer, and Williams, 2001; Bombardier et al., 2004; Krause et al., 2009; Krause, Reed, and McArdle, 2010; Fann et al., 2011); anxiety (Spielberger State-Trait Anxiety Inventory) (Spielberger et al., 1983); body appreciation (Functionality Appreciation Scale, FAS) (Alleva, Tylka, and Kroon Van Diest, 2017); and quality of life (World Health Organization Quality of Life Instruments, WHOQOL-BREF) (Jang et al., 2004). Participants self-reported on whether cardiovascular, bladder, bowel, and sexual functions were normal, abnormal, or absent (Autonomic Standards Assessment Form) (Krassioukov et al., 2012). Functional performance was assessed with the Spinal Cord Injury Functional Index (SCI/FI) (Slavin et al., 2016), across four domains: basic mobility, self-care, fine motor function, and ambulation (Keeney et al., 2018). Participants self-identified goals related to important daily life activities that were currently difficult to perform because of neuropathic pain (Patient-Specific Functional Scale, PSFS) (Westaway, Stratford, and Binkley, 1998).

### 2.5 Statistical analysis

#### 2.5.1 Sample size

Power and sample size calculations were made based on estimates from another body awareness therapy (i.e., cognitive multisensory rehabilitation) in adults with SCI (Van de Winckel et al., 2023b). Assuming the same SD estimate, *n* = 18 participants have >98% power to detect the same pain reduction of 2.31 points on the NPRS (*d* = 1.12) with a two-sided significance level of 0.05 using a paired *t*-test, and 80% power to detect a pain reduction of 1.46 points (*d* = 0.72). A 30% attrition rate would result in 80% power to detect a pain reduction of 1.85 points (*d* = 0.89).

#### 2.5.2 Statistical analysis

Per-protocol analyses were conducted for all outcomes for our secondary objective. We have added intent-to-treat analyses for the ANOVA tests and longitudinal modeling of the pain outcomes. We imputed the data with multiple imputations using the “mice” package in R, conducted the ANOVA test, Tukey’s *post hoc* comparisons, and the longitudinal modeling using linear mixed models for each imputed dataset, and averaged the results across all datasets.

The present data provide an estimate of key trial elements to determine whether to proceed to a larger randomized controlled trial. Quantitative variables are summarized using descriptive statistics at each time point. We used standard statistical software R version 4.2.1. Data from the first 12 weeks were analyzed using a repeated-measures ANOVA, with Tukey’s *post hoc* tests to evaluate changes between every pair of time points. The data at 1-year follow-up with missingness were compared to each of the time points during the 12-week main study using the paired t-tests with Bonferroni correction of the *p*-values.

## 3 Results

### 3.1 Demographic and behavioral data and feasibility measures

**FIGURE 2:**
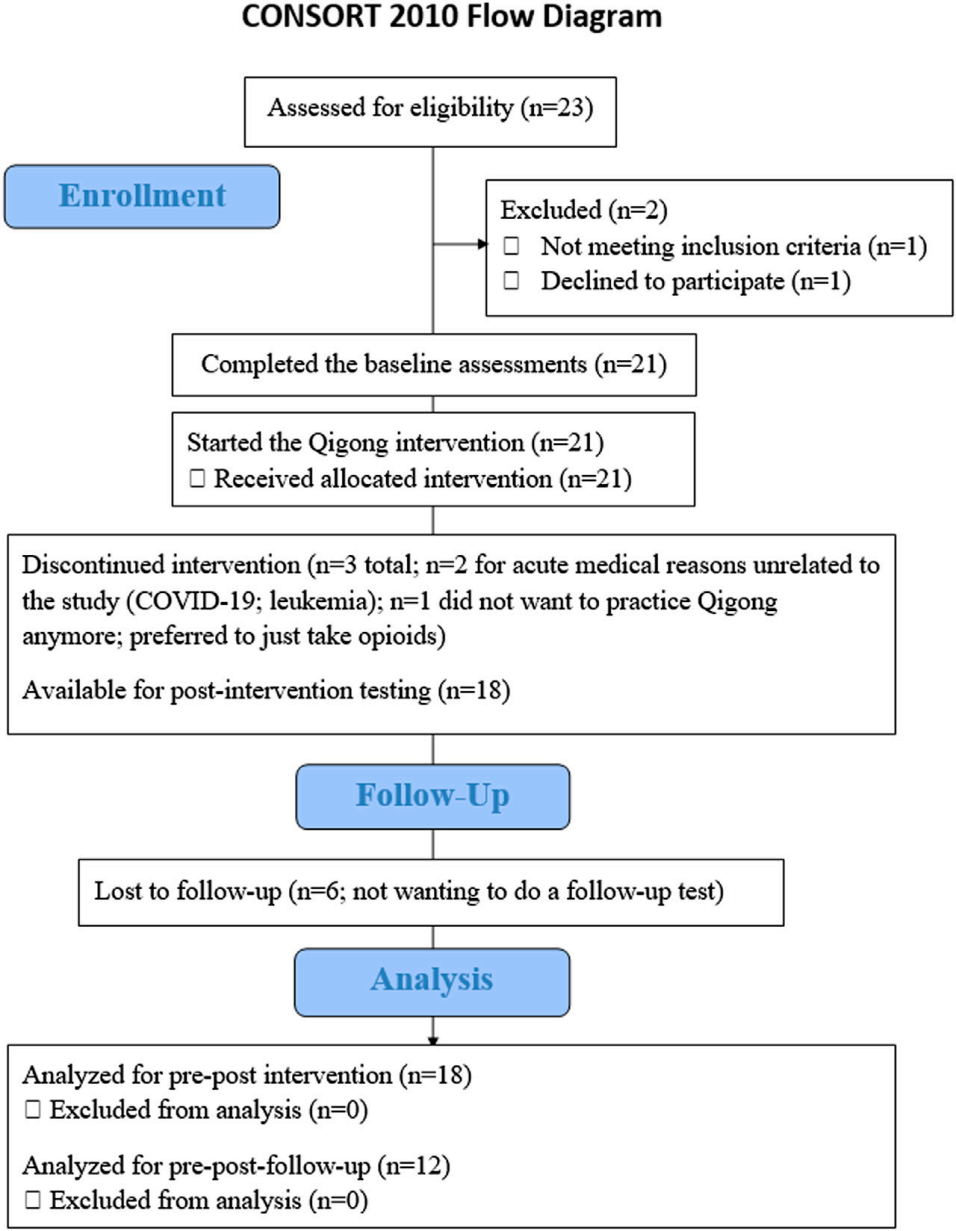
CONSORT flow diagram showing flow of participants throughout the study.

Figure 2 shows the CONSORT study flow chart of this non-randomized clinical trial. We recruited 23 adults with SCI between 1 July 2021 and 2 February 2023. We exceeded the *a priori* set feasibility benchmarks by retaining 18 participants (i.e., 82% retention) who completed the study, with 100% adherence to all study components, including the 6-week follow-up. Reconsenting (*n* = 12) at 1-year follow-up assessment was completed on 2 February 2023.

Table 1 displays the demographic and behavioral data of the participants. Among the 18 adults, six were women, 10 had paraplegia, and eight had tetraplegia; 33% of adults had a complete SCI (i.e., slightly below our estimated 40% recruitment). Mean age was 59.61 ± 11.54 years (range 30-76 years), and they were 15.17 ± 11.16 years post-SCI (1–40 years). The sex ratio of new SCI cases of men to women is approximately 4:1 (i.e., 78%). We enrolled a representative sample of women with SCI (33.33%), adults, 65+ years of age (38.89%), adults living in rural areas (33.33%), and veterans (16.67%). Despite recruiting nationally, all participants were non-Hispanic White people, pointing to the need for more diverse recruitment strategies. Approximately 67% of the participants had socioeconomic distress and were dependent on Medicare/Medicaid. Almost 17% of the participants were below the poverty threshold (Federal Poverty Guidelines - ProJusticeMN, 2023).

**TABLE 1:** Demographic and clinical characteristics of adults with spinal cord injury and neuropathic pain.

	Adults with SCI and neuropathic pain (n = 18)
**Age**, years mean ± SD (range)	59.61 ± 11.54 (30–76)
**Sex**, n (%)	
Male	12 (66.67)
Female	6 (33.33)
**Gender identity**, n (%)	
Male	12 (66.67)
Female	6 (33.33)
Others	0 (0.00)
**Ethnicity**, n (%)	
Hispanic	0 (0.00)
Non-Hispanic	18 (100.00)
**Race**, n (%)	
White	18 (100.00)
Asian	0 (0.00)
African-American/Black	0 (0.00)
Multi-racial	0 (0.00)
Hawaiian or other Pacific Islander	0 (0.00)
Others	0 (0.00)
**Veterans**, n (%)	3 (16.67)
Yes/no	15 (83.33)
**Location of residence**, n (%)	
Rural	6 (33.33)
City or suburb	12 (66.67)
**Financial situation**, n (%)	
Socioeconomic distress	12 (66.67)
Below the poverty threshold	3 (16.67)
**Baseline neuropathic pain intensity level**, mean ± SD	
High	7.94 ± 2.15
Average	4.78 ± 2.69
Low	3.06 ± 2.24
**Time since spinal cord injury**, years, mean ± SD, range (years)	15.17 ± 11.16 (1–40)
**Etiology of spinal cord injury**, n (%)	
Traumatic	13 (72.22)
Non-traumatic	5 (27.78)
**Spinal cord injury lesion level**, n (%)	
Cervical	8 (44.44)
Thoracal	7 (38.89)
Lumbar	3 (16.67)
Sacral	0 (0.00)
**AIS level**, n (%)	
A	6 (33.33)
B	1 (5.56)
C	6 (33.33)
D	5 (27.78)
**Level of impairment**, n (%)	
Paraplegia (complete; incomplete)	4 (22.22); 6 (33.33)
Tetraplegia (complete; incomplete)	2 (11.11); 6 (33.33)
**Ambulation, n (%)**	3 (16.67)
Walking without assistance	1 (5.56)
Combined use of walking with and without assistance	0 (0.00)
Walking with assistance	3 (16.67)
Manual wheelchair	2 (11.11)
Combined use of manual and motorized wheelchairs	1 (5.56)
Combined use of motorized wheelchair and walking with assistance	1 (5.56)
Combined use of manual wheelchair and walking with assistance	7 (38.89)
Motorized wheelchair	0 (0.00)
**Ambulation, mean percent time (SD)**	
Walking	21.11 (±40.85)
Walking with assistance	2.5 (±6.47)
Manual wheelchair	30 (±44.95)
Motorized wheelchair	46.39 (±48.62)
**Neuropathic pain medication at baseline**, n (%)	
Advil	1 (5.55)
Aspirin	1 (5.55)
Belbuca	1 (5.55)
Duloxetine	8 (44.44)
Gabapentin	1 (5.55)
Ibuprofen	1 (5.55)
Lamictal	1 (5.55)
Lidocaine patch	1 (5.55)
Lyrica	4 (22.22)
Naproxen	1 (5.55)
Oxycodone	1 (5.55)
Tegretol	1 (5.55)
Tylenol	1 (5.55)
**Mini-Mental State Examination-short version** (cutoff score <13/16), mean ± SD	15.06 ± 1.21
**Kinesthetic and Visual Imagery Questionnaire** (cutoff score <15/25 points), mean ± SD	23.44 ± 2.01

AIS level, International Standards for Neurological Classification of SCI American Spinal Cord Injury Association Impairment Scale (ISNCSCI-AIS) exam.

### 3.2 Outcome measures

For our primary objective, we exceeded our *a priori* benchmark of a minimum 70% of participants practicing at least 2x/week: group average was 169.72 min or 137.98% of the required Qigong practice intensity (3x/week). There were no study-related adverse events.

Participants almost unanimously reported that they could not wait to get back to the Qigong practice after the 6-week follow-up because they benefitted so much from it. At 1-year follow-up, one person was still performing Qigong every day. Five participants used Qigong when needed as a tool to bring their pain down. Two participants developed their own methods of breathing and mindfulness, based on what they learned in the program. Four participants had not performed Qigong since the end of the intervention period.

In total, 17 out of 18 participants (94.44%) were satisfied with the program (exceeding the 70% benchmark). One person [59-year-old man] was neutral and expressed: “I feel neutral about the Qigong exercises and weekly calls. I am pain-free now, and my goals (PSFS) were all achieved at week 6 and were still achieved at week 12.”

For our secondary objectives, all means and standard deviations of the outcome measures are listed in Table 2. The main findings are reported in the following paragraphs.

**TABLE 2:** Primary and secondary outcome measures in adults with SCI-related neuropathic pain at five time points.

Outcome measure	Baseline (mean ± SD)	6-week Qigong (mean ± SD)	12-week Qigong (mean ± SD)	6-week follow-up (mean ± SD)	1-year follow-up (mean ± SD)	Repeated-measures ANOVA (baseline–6wFU)	Significant Tukey’s *post hoc* pairs	Paired *t*-tests (6wFU–1YFU)
n	(*n* = 18)	(*n* = 18)	(*n* = 18)	(*n* = 18)	(*n* = 12)			
Highest NPRS	7.94 ± 2.15	5.94 ± 2.48	4.17 ± 3.07	4.39 ± 3.05	4.92 ± 3.03	F (3.15) = 19.70 *p* < 0.0001*	B–6wQ	t = 2.24 *p* = 0.05
							B–12wQ	
							B–6wFU	
							6wQ–12wQ	
							6wQ–6wFU	
Average NPRS	4.78 ± 2.69	3.92 ± 2.57	2.72 ± 2.54	2.94 ± 2.58	2.75 ± 2.01	F (3.15) = 7.65 *p* = 0.003*	B–12wQ	t = 1.74 *p* = 0.11
							B–6wFU	
							6wQ–12wQ	
Lowest NPRS	3.06 ± 2.24	2.00 ± 2.28	1.67 ± 2.45	1.61 ± 2.40	1.08 ± 2.19	F (3.15) = 8.24 *p* = 0.002*	B–6wQ	t = 2.25 *p* = 0.81
							B–12wQ	
							B–6wFU	
Interference on- Activity	5.39 ± 3.55	3.61 ± 2.38	1.94 ± 2.29	2.11 ± 2.37	2.67 ± 2.84	F (3.15) = 10.09 *p* = 0.0007*	B–6wQ	t = 1.91 *p* = 0.82
							B–12wQ	
							B–6wFU	
							6wQ–12wQ	
							6wQ–6wFU	
- Mood	5.00 ± 3.50	3.56 ± 2.23	1.61 ± 2.59	1.67 ± 2.22	1.92 ± 2.35	F (3.15) = 10.95 *p* = 0.0005*	B–6wQ	t = 2.24 *p* = 0.05
							B–12wQ	
							B–6wFU	
							6wQ–12wQ	
							6wQ–6wFU	
- Sleep	5.44 ± 3.57	3.94 ± 3.44	2.28 ± 3.04	2.44 ± 3.38	2.83 ± 3.95	F (3.15) = 8.55 *p* = 0.002*	B–12wQ	t = 1.43 *p* = 0.18
							B–6wFU	
							6wQ–12wQ	
Spasm- Frequency	1.67 ± 1.37	1.22 ± 1.35	0.50 ± 0.71	0.39 ± 0.61	0.50 ± 0.90	F (1.83, 31, 12) = 12.71 *p* = 0.0001*	B–12wQ	t = 1 *p* = 0.34
							B–6wFU	
							6wQ–12wQ	
							6wQ–6wFU	
- Severity	1.39 ± 1.14	1.06 ± 1.16	0.67 ± 0.84	0.33 ± 0.49	0.42 ± 0.67	F (3.15) = 7.59 *p* = 0.003*	B–12wQ	t = 1 *p* = 0.34
							B–6wFU	
							6wQ–12wQ	
PHQ-9	6.94 ± 5.54	5.22 ± 4.58	4.61 ± 4.82	4.39 ± 4.78	NA	F (3.15) = 3.48 *p* = 0.04*	B–12wQ	NA
							B–6wFU	
PSFS	1.07 ± 1.46	4.04 ± 2.59	7.76 ± 2.85	7.93 ± 2.91	7.64 ± 3.19	F (1.96, 33.35) = 53.18 *p* = 0.0001*	B–6wQ	t = −0.74 *p* = 0.47
							B–12wQ	
							B–6wFU	
							6wQ–12wQ	
							6wQ–6wFU	
State anxiety	35.00 ± 10.95	30.11 ± 9.79	29.67 ± 11.88	27.94 ± 7.61	NA	F (3.51) = 3.93 *p* = 0.013*	B–6wFU	NA
Trait anxiety	40.11 ± 10.71	35.78 ± 10.05	35.28 ± 11.60	34.39 ± 10.07	NA	F (3.51) = 5.79 *p* = 0.0017*	B–6wQ	NA
							B–12wQ	
							B–6wFU	
FAS	3.12 ± 0.58	3.13 ± 0.60	3.41 ± 0.60	3.52 ± 0.51	NA	F (3.15) = 5.81 *p* = 0.008*	B–12wQ	NA
							B–6wFU	
							6wQ–12wQ	
							6wQ–6wFU	
WHOQOL - Physical health	51.33 ± 19.57	58.44 ± 18.68	59.28 ± 17.40	61.28 ± 17.76	NA	F (3.51) = 4.69	B–12wQ	NA
						*p* = 0.006*	B–6wFU	
- Psychological health	64.61 ± 17.20	64.72 ± 18.20	69.61 ± 19.21	67.78 ± 18.13	NA	F (3.51) = 2.68		NA
						*p* = 0.057		
- Social relationships	53.17 ± 20.60	53.50 ± 21.16	59.72 ± 17.67	61.56 ± 17.76	NA	F (3.51) = 3.66		NA
						*p* = 0.018*		
- Environment	77.56 ± 16.76	79.56 ± 15.87	79.33 ± 19.78	81.67 ± 16.49	NA	F (3.51) = 0.84 *p* = 0.48		NA
Autonomic Function - General function	21.72 ± 3.18	21.72 ± 3.18	22.78 ± 1.83	23.00 ± 1.57	23.17 ± 1.53	F (3.51) = 5.137	B–6wFU	Same value
						*p* = 0.004*	6wQ–6wFU	
- Bladder function	1.94 ± 2.44	1.94 ± 2.44	2.22 ± 2.32	2.22 ± 2.32	2.83 ± 2.29	F (3.51) = 2.457	NA	t = 1.60
						*p* = 0.07		*p* = 0.14
- Bowel function	2.89 ± 2.45	2.89 ± 2.45	3.22 ± 2.46	3.22 ± 2.46	3.83 ± 2.17	F (3.51) = 2.83	NA	t = 1
						*p* = 0.05		*p* = 0.34
- Sexual function	2.28 ± 2.61	2.28 ± 2.61	2.50 ± 2.83	2.61 ± 2.85	3.33 ± 3.20	F (3,51) = 3.40	NA	Same value
						*p* = 0.03*		
SCI-FI para	(*n* = 10)	(*n* = 10)	(*n* = 10)	(*n* = 10)	(*n* = 9)			
- Basic mobility	26.40 ± 9.45	26.80 ± 9.81	31.40 ± 5.32	32.80 ± 4.05	31.22 ± 6.26	F (1.13, 10.14) = 10.54 *p* = 0.007*	B–12wQ	t = −1.20 *p* = 0.26
							B–6wFU	
							6wQ–12wQ	
							6wQ–6wFU	
- Self-care	26.80 ± 10.34	27.70 ± 9.09	30.20 ± 7.51	32.10 ± 5.28	29.78 ± 8.61	F (1.57, 14.10) = 8.87	B–12wQ	t = −1.07
						*p* = 0.005*	B–6wFU	*p* = 0.32
- Fine motor function	28.20 ± 4.02	28.90 ± 2.92	30.50 ± 1.90	31.40 ± 1.07	31.11 ± 2.03	F (1.76, 15.85) = 7.86	B–12wQ	t = −1
						*p* = 0.005*	B–6wFU	*p* = 0.35
- Ambulation	5.60 ± 6.93	6.30 ± 7.29	6.80 ± 7.51	7.30 ± 7.82	5.33 ± 7.71	F (1.79, 16.09) = 3.87	NA	t = −1.79
						*p* = 0.05		*p* = 0.11
SCI-FI quad	(*n* = 8)	(*n* = 8)	(*n* = 8)	(*n* = 8)	(*n* = 3)			
- Basic mobility	12.00 ± 11.26	13.63 ± 12.19	15.00 ± 12.82	15.50 ± 12.60	17.67 ± 15.04	F (3.5) = 4.03	NA	Same value
						*p* = 0.08		
- Self-care	14.00 ± 13.15	14.25 ± 12.71	17.38 ± 13.00	18.00 ± 13.02	22.67 ± 10.60	F (1.35, 9.44) = 7.03	B–12wQ	t = −2
						*p* = 0.02*	B–6wFU	*p* = 0.18
- Fine motor function	16.63 ± 10.34	17.25 ± 10.91	18.00 ± 10.80	18.25 ± 10.87	25.67 ± 5.03	F (3,5) = 2.30	NA	t = −1
						*p* = 0.19		*p* = 0.42
- Ambulation	4.00 ± 6.30	4.50 ± 6.89	4.63 ± 6.93	4.75 ± 7.19	4.33 ± 7.51	F (1.34, 9.37) = 1.63	NA	Same value
						*p* = 0.24		

12wQ, 12 weeks of Qigong practice; 6wQ, 6 weeks of Qigong practice; 6wFU, 6-week follow-up; B, baseline; NPRS, Numeric Pain Rating Scale (used here to assess neuropathic pain intensity in the prior week); average NPRS, pain experienced most of the time in the past week; PHQ-9, Patient Health Questionnaire-9; PSFS, Patient-Specific Functional Scale; FAS, Functional Appreciation Scale; SCI/FI, Spinal Cord Injury Functional Index; WHOQOL, World Health Organization quality-of-life scale. Significant *p*-values (*p* < 0.05) are indicated with an *.

The results with repeated-measures ANOVA (Table 2) showed a significant reduction in the highest, average, and lowest neuropathic pain levels from baseline to 12 weeks of Qigong, which was maintained at the 6-week (*n* = 18) and 1-year follow-ups (*n* = 12). Some participants had urinary tract infections or underwent surgeries at 1-year follow-up, causing a temporary increase in the highest neuropathic pain levels. The highest, average, and lowest pain all showed a significant reduction over time for both intent-to-treat and per-protocol analyses. The ANOVA and Tukey’s *post hoc* comparisons showed a significant reduction in pain pre–post Qigong practice, and no significant changes were observed between post-Qigong and 6-week follow-up.

**FIGURE 3:**
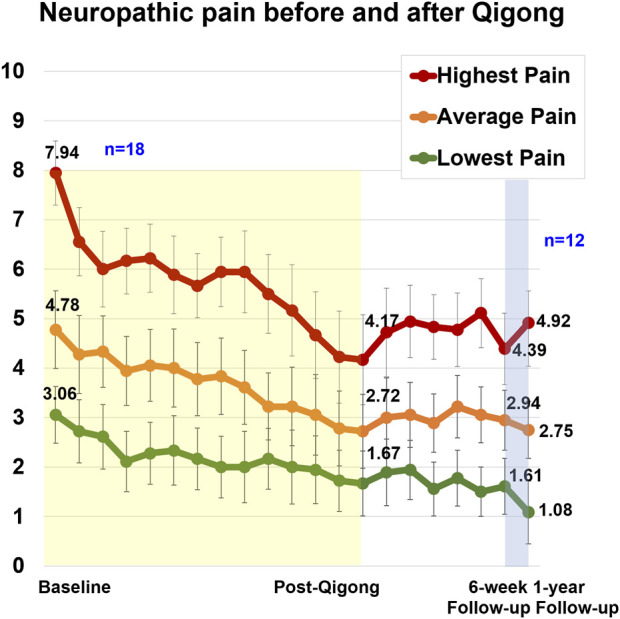
Weekly neuropathic pain levels for highest, average, and lowest pain in 18 adults with SCI. The 12-week Qigong training is highlighted in yellow. During the 6-week follow-up (*n* = 18), participants did not practice Qigong but implemented body awareness in daily life. Between the 6-week and 1-year follow-ups, participants could restart the Qigong practice at the frequency of their choice (*n* = 12).

The weekly highest, average, and lowest neuropathic pain intensity ratings (NPRS) (Hanley et al., 2006) are shown in Figure 3. The highest *neuropathic pain level* at baseline was 7.94 ± 2.33. After 12 weeks of Qigong, there was a reduction of 3.78 points (large effect size, Cohen’s *d* = 1.75), 2.06 points (*d* = 1.12), and 1.39 points (*d* = 1.08) for highest, average, and lowest neuropathic pain levels, respectively, exceeding a minimal clinically important difference for the highest and average neuropathic pain levels (>1.80 points) (Williamson and Hoggart, 2005; Hanley et al., 2006; Galhardoni et al., 2019; Kim et al., 2020). After 12 weeks of Qigong, four participants were pain-free, and nine participants scored 0 on their lowest neuropathic pain. Pain reduction was maintained over the 6-week follow-up period (three were completely pain-free, and nine participants scored 0 on the lowest neuropathic pain). The person who was pain-free after 12 weeks of Qigong but not at the 6-week follow-up reported that the increased pain was due to the ongoing bladder issues and that his highest pain occurred infrequently. In total, 15 out of 18 participants reported significant pain reduction. Those that did not report any pain reduction shared that they enjoyed “listening” or “watching” the video but did not perform the kinesthetic imagery or did not apply the kinesthetic imagery tools in daily life or had significant pressure ulcers. Pressure ulcers or urinary tract infections during the study could cause temporary increases in neuropathic pain. However, even those three participants reported that they enjoyed watching the video and moving; they had fewer spasms, better sleep, and it was relaxing and calming.

**FIGURE 4:**
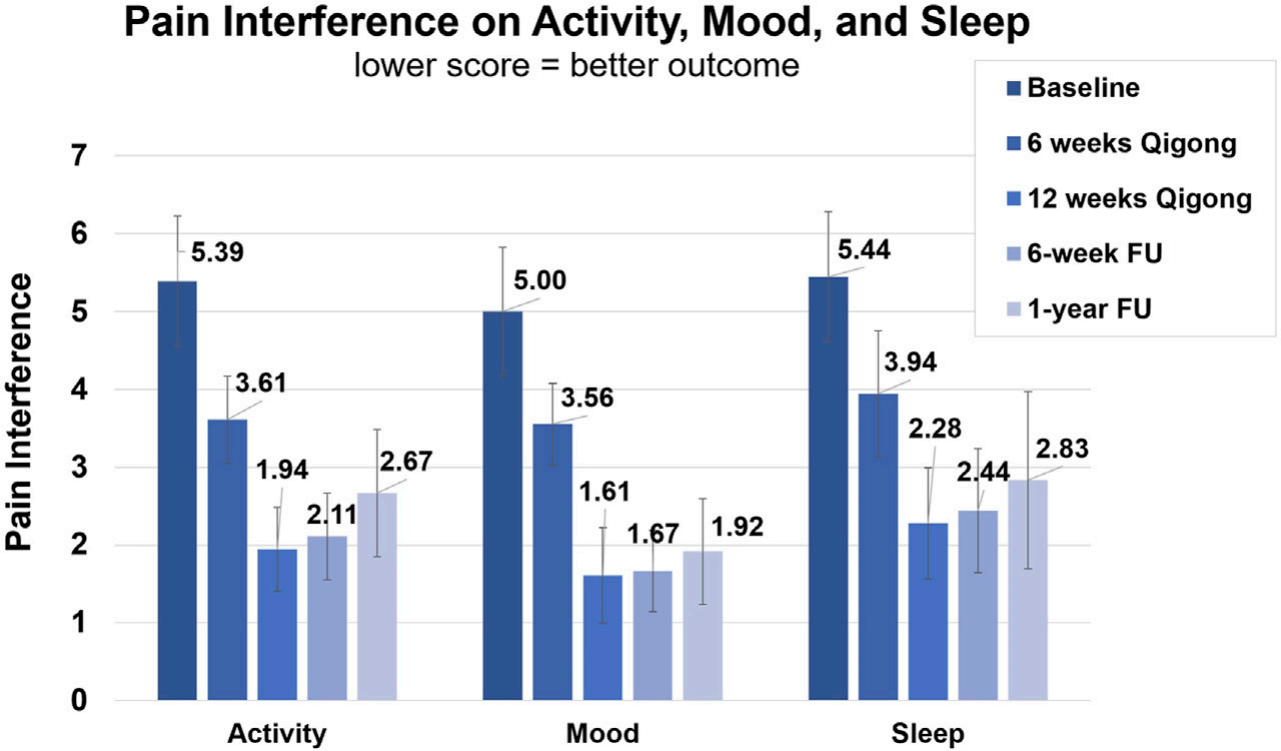
Clinical assessments of the NINDS-CDE International SCI Pain Basic Data Set Version 2.0.1. Average score of interference of neuropathic pain with activity, mood, and sleep. Assessments were taken at baseline, 6-week Qigong practice, 12-week Qigong practice, 6-week follow-up, and 1-year follow-up.

**FIGURE 5:**
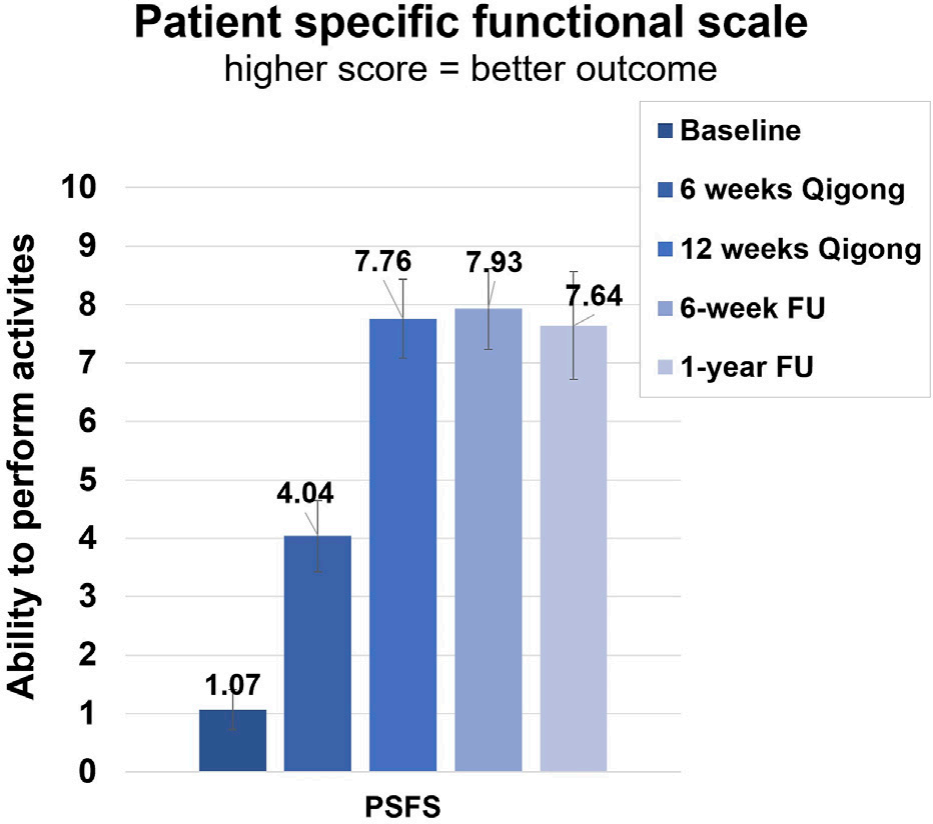
Clinical assessments of the Patient-Specific Functional Scale (PSFS). Average score of ability to perform three functional activities. Assessments were taken at baseline, 6 weeks of Qigong practice, 12 weeks of Qigong practice, 6-week follow-up, and 1-year follow-up.

After 12 weeks of Qigong, participants also reported reduced spasm frequency (change score 1.17 ± 1.20, *d* = 0.98) and severity (0.72 ± 1.02, *d* = 0.71), and reduced interference of neuropathic pain on activity (3.44 ± 2.53, *d* = 1.36), mood (3.39 ± 2.40, *d* = 1.41), and sleep (3.17 ± 2.77, *d* = 1.14, Figure 4 1). Participants performed better on functional activities (PSFS, 6.68 ± 3.07, *d* = 2.18, Figure 5), had reduced anxiety (state anxiety, −5.33 ± 11.84, *d* = 0.45; trait anxiety, −4.83 ± 6.19, *d* = 0.78), improved mood (2.33 ± 3.31, *d* = 0.70), body appreciation (−0.29 ± 0.48, *d* = 0.60), and quality of life (physical health, 7.94 ± 10.59, *d* = 0.75; psychological health, 5.00 ± 9.13, *d* = 0.55; social relationships, 6.56 ± 11.27, *d* = 0.58; and environment, 1.78 ± 7.06, *d* = 0.25). Overall, participants reported more intense daily activity (e.g., reorganizing furniture). One person could play the piano again after 3 years for 30 min/day, which is his favorite hobby. These significant improvements were maintained at follow-ups.

The results from the *post hoc* tests showed that cardiovascular function improved between baseline and 6-week follow-up and was maintained at 1-year follow-up, and participants’ reports confirmed that this was related to improved temperature regulation. Four participants could better feel the need for bowel movements (greater interoception); they had improved sphincter control and were better at avoiding leakage. Four other participants could feel and enjoy sexual activity more, which was reflected by improvements in sexual function on the Autonomic Standards Assessment Form. Two participants reported increased awareness of the need to urinate or feeling urine passing through the catheter. Several participants became more aware when something was “off” in their bodies, and thus they could address urinary tract infections or uncomfortable seating positions quicker.

Finally, participants with paraplegia reported improved balance, which was reflected in improved scores in basic mobility, self-care, and fine motor function on the SCI/FI assessment. An example of improved fine motor function was that they did not need to use their hands anymore for balance and, thus, could do fine motor activities while sitting in the wheelchair. Participants with tetraplegia improved in self-care.

## 4 Discussion

The results of this pilot non-randomized controlled trial demonstrate feasibility and adherence to a remote Qigong intervention and study protocol. Qigong practice was well tolerated with no study-related adverse events. The vast majority of participants enjoyed Qigong practice and continued practicing as needed afterward, pointing to a change in behavior that helped them gain control over an otherwise debilitating symptom. The feedback from some of the participants after the Spring Forest Qigong practice can be found in the following paragraphs:

[65-year-old woman] “It got me back in touch with meditating that I felt I was missing in my life. At times, I have more body awareness which can further increase over time. I have much less pain. I am grateful and glad that I was able to do it.”

[68-year-old man] “The fact that the pain is reduced makes life more pleasurable, especially with social contacts (family gatherings).”

[53-year-old man] “Qigong is relaxing and calming. That relaxed and calm feeling carries over into the day.”

[76-year-old man] “Two thumbs up. I can’t say enough good about it, how many good things it is doing for me. I am practicing almost every day. Before the study, I was ready to jump from a building (“ready to get out of this body”) and now I do not have these feelings anymore. The underlying results are excellent: pain-free, no spasms, sleep is good now, calmer, can handle things better. I can play the piano again after 3 years. Before the study, I was antsy after about 15 min at the computer, and I had to take a nap on the bed. Now I can sit at the computer for 3–4 h, and it feels good. At night, I used to be cold and needed a sweatshirt to sleep. Now I stay comfortably warm, and I do not need the sweatshirt anymore. In the last 2 weeks, I also regained sensation of the catheter. I feel when urine is passing through which I could not feel before.”

[46-year-old woman] “It was really helpful to get more in tune with my body. My pain is down, and I am sleeping better. I knew that if I signed up for the study I would do it because of outside accountability. There was curiosity involved. The Qigong was relaxing and soothing. It was helpful for my children as well. The plantar fasciitis is better. It is healing faster. The tingling in the feet is better, and the pain is down so I am sleeping better. I am more social; I have more energy and organize things easier. I have more control over life. It is easier now for me to make decisions and to undertake a big thing like a move.”

[58-year-old man] “I am over the moon! I never thought that it would go this well. Before the study, I was spending much time in the shower to deal with the pain and now, I have had no need at all anymore for that. My feeling has been that it was absolutely wonderful! I am so happy for the opportunity to be part of this study because it made so much difference. I do not need to lay in the shower anymore to reduce the pain, and I was able to sit for a whole day inspiring students and researchers by sharing my journey, telling them: And here is this study … what a difference it is making in people’s lives. The idea that there is something out there that could help me, considering how long ago I had the SCI (29 years of dealing with this pain!). . . and here in only 8 weeks it makes a profound difference in my life. It gives people hope. Prior to the study, I would not have been able to sit here and talk to you for so long. This is another proof of how profound the difference is that this study has made.”

[60-year-old woman] “What you practice grows stronger. It stayed with me. The positivity part has been more prominent there for me. My life is changing a lot. I have been able to realize that those five emotions from the Five Element Qigong Healing Movements are what I want for my life. There are changes in friendship. I have stopped taking calls from negative people and spent more time with positive people. As simple as those points are, they are profound. Years ago, a therapist told me that you can decide in the morning how you want to use your energy. That makes sense now. I have been using energy now to bring joy and contentment. I have been a lot more active, I do not have to lay down anymore during the day.”

Specific exercises that participants preferred were the bouncing (at the beginning of the video), the focus on positive emotions, and movements 2 and 5. Some participants liked the second movement because of the focus on the vertical alignment and flow in the body (head to toe) while gently moving the arms in an elliptical movement up and down and the fifth movement for the connection with the gratitude feeling and heart’s energy. They also reported that the kinesthetic imagery, e.g., imagining the feeling of how the foot would leave imprints in the sand or being aware/imagining the weight shift in the pelvis during transfers and at other moments (e.g., during sitting in the wheelchair) helped with reducing the pain.

Twelve weeks of Qigong practice appears to be the best duration for sustained neuropathic pain reduction (Table 2). The participants reported that the video was easy to follow and understand. The Qigong practice frequency is feasible given that the majority of participants practiced more often than the requested 3x/week. The adaptation to the Qigong practice for adults with SCI by combining active movements to their ability level with kinesthetic imagery seemed particularly an effective and worth pursuing further in a larger study.

In addition to pain reduction and improvements in mood and function, participants reported a change in *mindset* in how they approached their pain. At baseline, participants distracted themselves from pain or ignored pain, and as a result, the pain worsened often. After Qigong practice, many participants shared that they learned how to connect with their body, developed the ability to listen to their bodies, and understood when their bodies gave signals of an uncomfortable position or situation (e.g., bowel or bladder issue). Participants felt they reacted quicker to these signals, and the uncomfortable feeling did not develop into pain. Examples include that participants more quickly repositioned themselves in the chair to avoid pressure sores, or participants were able to identify quicker when they had a urinary tract infection or that “something was off” in the body, prompting them to get medical help and treatment (e.g., detecting iron deficiency and receiving iron supplements). Events like urinary tract infections could cause short neuropathic pain flare-ups. However, given that chronic neuropathic pain is persistent and refractory to medications, the observed long-standing pain reduction all through the study is very likely related to the Qigong practice. Additionally, we observed in another study on adults with spinal cord injury that chronic neuropathic pain does not change when only medication (treatment-as-usual) is given (Van de Winckel et al., 2023b). Yet, our results need to be investigated in a future randomized controlled trial with a greater sample size.

There are different concepts around mindset. One concept distinguishes a growth mindset [i.e., believing that qualities can be cultivated and expanded through personal investment (Thames and Webster, 2015)] from a fixed mindset (i.e., believing that change is a threat). Another concept distinguished “stress-is-enhancing” mindset (i.e., stress leads to better performance, productivity, health, wellbeing, learning, and growth). which is also related to the ability to positively reframe a situation, greater wellbeing, and fewer depressive symptoms, from “stress-is-debilitating” mindset (Grünenwald et al., 2023). The latter (stress is debilitating) has been reported more frequently in adults with chronic pain vs. those without chronic pain. Similarly, views of coping with the pandemic (it is an opportunity vs. it is manageable vs. it is a catastrophe), where the first two groups (i.e., opportunity and manageable) reported greater wellbeing than those who viewed COVID-19 as a catastrophe (Zion et al., 2022).

While we did not specifically evaluate the type of mindset through questionnaires, the fact that the study used volunteer sampling might have attracted adults who were willing to try something new. Most of our participants had not heard of Qigong prior to entering the study, and some were skeptical about the ability of Qigong being able to help reduce pain, but they were willing to invest their time and explore the effects, given the debilitating situation of the neuropathic pain. Their skepticism often evaporated and their enthusiasm grew after the first effects of pain reduction were perceived, and they experienced an increased feeling of control over their body and confidence about being able to successfully do something to relieve the pain.

The increased body awareness and ability to listen to body cues, as well as being calmer, having better mood, better sleep, and being able to deal with things better were consistently reported among the participants. They also reported having more in control over their life, having more energy, and more confidence knowing that they now had tools to deal with the pain if the pain would come up again. They reported having a calmer mind and a calm body, feeling more peace and restful, more connectedness/in tune with the body. They figured out more quickly when something was not right. They experienced more happiness, and were ready to interact socially again, changing the circle of friends if needed by seeking out those with a positive outlook on life, or they reported taking on life goals or bigger house tasks such as rearranging furniture, or bidding on a house, or moving. One person reported it is easier now to make decisions and undertake big things, like a move. They reported that also other types of pain (e.g., plantar fasciitis or elbow tendonitis) got better. One participant reported her children enjoyed doing the Qigong with her. They also felt that the positive emotions and gratitude practiced during Qigong carried over into daily life.

There are some limitations to this study. The volunteer sampling may have led to selection bias of those that chose to invest in a 12-week program and thus may limit generalization. This non-randomized pilot clinical trial was conducted in a small sample, and thus validation in a larger sample with a randomized clinical trial design is needed. Even though we did not have a control group in this study, we know from our other studies with a control group of adults with spinal cord injury with similar intensity levels of neuropathic pain (*n* = 14) that 6 weeks of standard of care only (i.e., neuropathic pain medication) did not change their neuropathic pain levels or any other SCI-related measures (Van de Winckel et al., 2023b). We are therefore confident that the proposed results are due to Qigong and not due to the placebo. Moreover, neuropathic pain is known to be resistant to pharmacological and non-pharmacological interventions such as surgery, neurostimulation (Forte et al., 2022), and physical and psychological therapy, and therefore the large effect size results seen with Qigong are very encouraging. Furthermore, while we recruited participants living in remote areas and/or in financial distress, diversity in terms of race and ethnicity was missing. For example, adults of the Hispanic background account for 17.4% of the US population, or 55.4 million, (Velasco-Mondragon et al., 2016), and represent 8.3% of all SCI since 2005 (Spinal Cord Medicine, 2011; Spinal Cord Injury Statistics, 2016). Yet, they are underrepresented in SCI rehabilitation studies (National Spinal Cord Injury Statistical Center, 2022).

In conclusion, our pilot data demonstrate the feasibility and acceptability of practicing Qigong in adults with SCI-related neuropathic pain, generating promising results in terms of neuropathic pain and SCI-related symptoms. The data from the present work will inform the design of future randomized controlled trials. The remote delivery of Qigong offers multiple applications for use in the home or community. Further studies in adults with SCI of different races and ethnicity and Qigong delivery in other languages are needed.

## Data Availability

The datasets presented in this study can be found in online repositories. The names of the repository/repositories and accession number(s) can be found at the Dryad repository: doi: 10.5061/dryad.6t1g1jx43.

## Abbreviations

FA, Functionality Appreciation Scale; IRB, Institutional Review Board; NINDS-CDE, Neurological Disorders-Common Data Elements; NPRS, Numeric Pain Rating Scale; PHQ-9, Patient Health Questionnaire-9; PSFS, Patient-Specific Functional Scale; SCI, spinal cord injury; SCI/FI, Spinal Cord Injury Functional Index; WHOQOL-BREF, World Health Organization Quality of Life Instruments.
